# An enabling Framework for Blockchain in Tourism

**DOI:** 10.1007/s40558-022-00229-6

**Published:** 2022-08-08

**Authors:** Sreejith Balasubramanian, Jaspreet Singh Sethi, Shalini Ajayan, Cody Morris Paris

**Affiliations:** 1 Middlesex University Dubai, Dubai, United Arab Emirates; 3 Mohammed bin Rashid School of Government Dubai, Dubai, United Arab Emirates; 4 School of Tourism and Hospitality, Department of Tourism, University of Johannesburg, Johannesburg, South Africa

**Keywords:** Cryptocurrency, Automation, Intelligent environments, Digitalization, Disintermediation, Smart tourism, Blockchain technology, COVID-19

## Abstract

This viewpoint article proposes an enabling framework that identifies the use of various blockchain technologies in tourism and their applications (digitalization, automation, disintermediation, and intelligent environment) across the different stages of travel (pre-trip, during the trip, and post-trip). As we know, the tourism sector contributes immensely to world GDP and job creation. However, the COVID-19 pandemic, even after two years since it first appeared, continues to adversely impact the tourism prospects of countries across the world due to nationwide lockdowns and travel restrictions. As the world tries to adapt to the “new normal,“ the tourism sector is forced to re-think its ways of doing business and bring about innovations to facilitate the new norms of contactless and safe transactions. Also, the sector, more than ever, need to effectively deal with its inherent challenges such as transparency and credibility of information, fraudulent practices, opportunistic behavior of intermediaries, and foreign currency risks. Blockchain technology can transform the tourism sector by offering innovative solutions that address its pressing issues. However, our current understanding of blockchain application in tourism is quite limited, with previous work being largely fragmented and narrow in terms of both scope and application. We foresee that the insights offered in this viewpoint, including the framework, will advance both theory and practice and facilitate the implementation of blockchain-enabled solutions across different travel stages.

## Introduction

The tourism sector is one of the significant contributors to the global economy. Before the pandemic, travel and tourism accounted for 10.4% of the global GDP (US$9.2 trillion) and 10.6% of all jobs in 2019 (WTTC 2021). The impact of COVID-19 on the tourism sector has been devastating, particularly for countries dependent on tourism, with travel bans, international border closures, isolation, and social distancing measures. In 2020, the tourism sector suffered from a staggering loss of US$4.5 trillion in comparison to 2019, and its contribution to global GDP declined to 5.5% (WTTC 2021). International tourist arrivals declined by almost 74% over the previous year (UNWTO 2021), and tourist spending decreased by approximately 70%, resulting in losses of around 62 million jobs in 2020 (WTTC 2021; Abbas et al. 2021). It is clear from these figures that COVID-19 has severe economic consequences and continues to threaten the financial stability of the tourism sector (UNCTAD 2021).

Most countries have implemented strategies and measures, including technology-driven ones, to revive the tourism economy during COVID-19 and stimulate the sector’s recovery (OECD 2020). Technology-driven measures include digital payments, touchless service delivery, digital vaccination records, and crowd control technologies. However, the increase in digital technologies, automation, and online transactions has also aggravated the risk of misuse of personal information, privacy issues, cyber-attacks, and financial fraud (Cruz-Cárdenas et al. 2021).

Blockchain technologies can substantially transform the tourism sector (Treiblmaier et al. 2020). It is an “immutable distributed ledger” decentralized with no central authority and records are all validated as discrete and encrypted digital data events and transactions executed or shared among participants in a network (Irannezhad and Mahadevan 2021). It provides transparent, secure, trustworthy, and interoperable solutions, either as a standalone technology or integrated with other technologies (Balasubramanian et al. 2021a). Blockchain, therefore, is a natural fit to overcome the problems brought by the COVID-19 pandemic and the inefficiencies inherent to the tourism sector, such as transparency and credibility of information, fraudulent practices, opportunistic behavior of intermediaries, and foreign currency risks (Leal et al. 2021). For example, blockchain can enhance the trustworthiness and reliability of digital vaccination records. Further, blockchain could integrate the highly fragmented tourism value chain, including several actors, contracts, and transactions.

The foundation for this viewpoint and the framework presented was a systematic review of the existing blockchain tourism literature. Our systematic review using the search terms “Blockchain and Tourism” OR “Block chain and Tourism” (extraction date – 1st April 2021) in the Web of Science database initially identified 45 articles. After our screening protocols, we narrowed it to 26 relevant articles, and after a thorough synthesis, additional pertinent studies were identified and reviewed from the references cited. In addition, considering the still relevant novelty of the topic, various industry sources, including websites, magazines, reports, and news articles of leading consulting firms, governments, and global organizations, were reviewed to enrich our understanding of blockchain applications in tourism.

We made several initial observations upon reviewing this nascent and still fragmented literature. First and foremost, most of these studies are narrow in scope (rightfully so), focusing on one or a few specific blockchain applications; for example, Treiblmaier et al. (2020) have focused on cryptocurrencies, while Veloso et al. (2019) study focused on crowdsourcing. Other studies have focused on blockchain applications in specific domains of tourism, such as medical tourism (Pilkington 2020; Balasubramanian et al. 2022) or sustainable development in tourism (Tham and Sigala 2020). Many of the studies we have reviewed offer useful insights into specific applications, cases, or contexts; however, there has yet to be a systematic effort to understand the various use of blockchain technology across the lengthy tourism value chain (which begins before a tourists’ search for information and continues beyond their return home). Given the importance of the future of blockchain for tourism, this viewpoint provides a timely contribution to support research, practice, and policy needed for the large-scale acceptability and implementation of blockchain in tourism. The enabling framework for blockchain in tourism we present highlights blockchain-enabled solutions and their applications across the different stages of the tourist experiences.

## Development of the blockchain framework for tourism

**Fig. 1:**
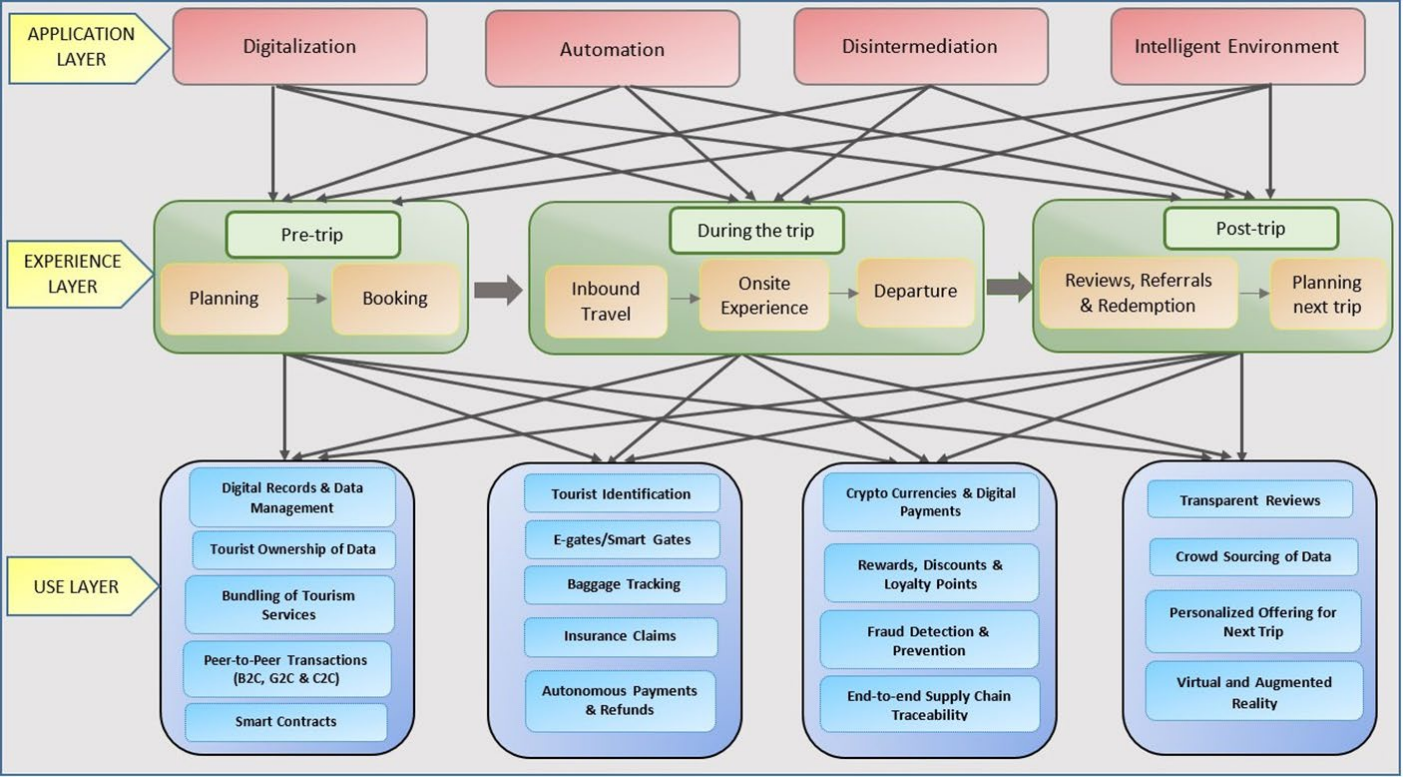
Blockchain Framework for Tourism

Figure 1 presents a visual representation of our enabling framework for blockchain applications in tourism. Central to our framework is the ‘Application Layer’ of blockchain in tourism, indicating the meaningful and managerially relevant categories of blockchain applications (digitalization, automation, disintermediation, and intelligent environment). The ‘Experience Layer’ presents the three stages of the tourist experience. During the pre-trip stage, tourists decide where to go, how to get there, and where to stay, while during the trip, tourists choose where and what to eat or what activities to engage in. During the post-trip (evaluation) phase, tourists express varying degrees of satisfaction which they share (Lee et al. 2020). This post-trip evaluation in reviews, blogs, and social posts is critical for inspiring other tourists in their pre-trip stage. The ‘Use Layer’ layer of the framework represents the uses of blockchain solutions in tourism (e.g., smart contracts, baggage tracking) across the different travel stages. Identifying these uses was a significant undertaking, given that the central task of developing any technology-enabled framework is identifying key technology solutions for inclusion (You and Feng 2020; Balasubramanian et al. 2021b, 2022).

This application layer (top layer) connects the experience layer (middle layer) with the use layer (bottom layer). The interconnections in the framework between the application, experience, and use layers illustrate that all three tourist travel stages can benefit from blockchain solutions that foster digitalization, automation, disintermediation, and intelligent environment. For instance, the tourism sector can benefit from digitalization (e.g., digital payments) across all three travel stages. Cryptocurrencies and digital payments contribute to the tourism sector’s digitalization drive by converting the physical ecosystem to a digital ecosystem, and this ‘digitalization’ may have different implications at different stages of the travel experience.

### Applications of blockchain: digitalization, automation, disintermediation, and intelligent environment

#### Digitalization

Digitalization uses digital technologies such as blockchain to convert the physical ecosystem to a digital ecosystem and then manage it virtually. It is changing the way people live, work, travel, and do business, and in the process, transforming and reshaping tourism (OECD 2020). As evident from the review, blockchain can enhance the digitalization drive of the tourism sector on a global scale and, in the process, improve the tourist experience and generate new business opportunities for the sector. For instance, blockchain solutions such as digital identity management, digital payments, and smart contracts have significantly reduced the inefficient paper-intensive processes seen in the tourism sector. Blockchain solutions have also increased transparency and minimized fraud and errors, processing times, and transaction costs for all parties involved (Kizildag et al., 2019). Blockchain can support governments’ drive towards cashless economies (Önder and Gunter 2020). Further, blockchain promotes contact-less payments, which is strongly encouraged during the COVID-19 pandemic (Önder and Gunter 2020). Therefore, international travelers could benefit from having a unified cashless and contactless payment system without the need to carry foreign currency cash. Finally, increasing P2P transactions via blockchain can further advance the sharing economy (Filimonau and Naumova 2020; Tham and Sigala 2020).

Many governments, especially those relying heavily on tourism (e.g., Malta, the Caribbean economies, Aruba, and the Marshall Islands), have started to make significant investments in blockchain technology to enhance their tourism sector. Also, there are several blockchain-backed virtual tourism solutions. For tourists, who cannot travel due to COVID-19 travel restrictions, virtual reality allows them to virtually visit and experience destinations from the comfort of their homes (Entrepreneur 2021). For example, Victoria VR is a blockchain-based ecosystem that unites multiple virtual reality platforms and creates a world filled with user-created photorealistic content recorded in the blockchain. Therefore, Victoria VR users can work, visit tourist destinations, participate in meetings, enjoy concerts and play games without any risks or need to travel (Entrepreneur 2021). Further, the virtual reality and augmented reality solutions powered by blockchain technology allow verified users to virtually participate in sold-out physical events such as music concerts by purchasing tokens (Mofokeng and Matima 2018). Given the significant investments in the Metaverse, we would expect blockchain applications supporting the tourism metaverse to be a key consideration in the near future.

#### Automation

Tourism process automation on a global scale can be significantly driven by or facilitated by blockchain, thereby increasing the sector’s efficiency, accuracy, and productivity. For instance, blockchain can automate a range of business dealings or transactions between tourists and service providers, including insurance pre-approvals, without human intervention from a legal perspective (Shen and Bai 2020). Blockchain-powered smart contracts are self-executing and self-enforcing, with pre-defined rules, procedures, and penalties (Erceg et al. 2020; Irannezhad and Mahadevan 2021). For example, blockchain offers automatic flight delay insurance for its customers; as soon as a flight delay is detected, compensation is initiated immediately and securely, thus avoiding additional paperwork (Radanović and Likić 2018). Similarly, blockchain can automate secure check-ins at airports (e.g., digital passports, smart gates) and hotels through digital keys or biometric identification (Thees et al. 2020). This automation creates mutually beneficial outcomes for tourists and service providers by reducing processing times.

#### Disintermediation

Blockchain’s ability to increase the level of disintermediation (reduced need for intermediaries) is evident. Blockchain applications can remove intermediaries such as online travel agents from the tourism value chain (Önder and Treiblamer 2018). Disintermediation enables tourists to make informed decisions and also makes travel more affordable. It shifts the balance of power from institution-centric to tourist-centric mode. Disintermediation increases accountability, transparency, trust, and collaboration among stakeholders in the tourism sector (Rashideh 2020). For example, digital payments using cryptocurrencies eliminate the need for a central authority such as banks or other intermediaries (Valeri and Baggio 2021). Also, blockchain helps tourism firms, especially smaller firms, to gain access to consumer data; they can buy data directly from the consumers rather than relying on and buying from Facebook and Google, which sells data to “anyone” who can afford to pay for it (Tham and Sigala 2020). Disintermediation also minimizes the privacy and security concerns of data theft, identity theft, and credit card theft, that tourists may have about sharing their sensitive data, including financial data, with travel agencies or other intermediaries (Çapar 2020). These concerns are reduced because blockchain is a ‘privacy-by-design solution and will give data access only to authorized actors after verifying the identity, such as by using a digital signature or certificate. It also provides more control and ownership for tourists over the personal data they share with service providers (Tyan et al. 2020).

#### Intelligent Environment

Blockchain technologies have the potential to make the sector more intelligent. For instance, advances in blockchain combined with big data analytics and artificial intelligence can enhance understanding of tourist needs, enabling personalized recommendations of tourism products and services (Leal et al. 2021). Similarly, blockchain combined with biometric readers can allow tourists to go through automated clearance gates and hotel check-in, significantly eliminating identity theft and fraud (Nam et al. 2021; Tyan et al. 2020). Likewise, blockchain combined with the Internet of Things (IoTs), RFIDs, and QR codes can provide end-to-end supply chain visibility, reducing counterfeit and fake products and services. For example, blockchain can provide end-to-end supply chain traceability of wine, from grape cultivation to winemaking and distribution/sales until the final stage of consumption at the cellar door or restaurant (Tham and Sigala 2020).

### Blockchain solutions across stages of the tourist experience

In the following sections, we explore some of the challenges and inefficiencies that can be addressed through blockchain solutions. We have organized this discussion into pre-trip, during-trip, and post-trip stages of travel.

#### Pre-trip stage

One of the challenges of the tourism sector is the lack of transparency, such as information on hotel’s capacities, different rates at different source markets, and data discrepancies due to bookings passing through multiple systems, human errors, double booking, manual and paper-based communication (Irannezhad and Mahadevan 2021). It is estimated that data discrepancies can impact about 5-10% of bookings (around US$10 billion) due to the volume of bookings passing through multiple systems (Microsoft 2016). Blockchain provides various solutions to enhance transparency and minimize data discrepancies. First, it eliminates the need for manual or paper-based communications and payments (Irannezhad and Mahadevan 2021; Leal et al. 2021). Blockchain provides a one-stop-shop for tourists to connect and coordinate tour and travel activities, improving their user experience and security where all price information and other aspects are continuously updated (Nam et al. 2021; Irannezhad and Mahadevan 2021). It enables tourists open access to real-time information on availability and prices to find and book the best deals, enabling improved itinerary planning (Önder and Gunter 2020; Tyan et al. 2020). It also reduces overbooking and manipulation of booking and prices (Nam et al. 2021; Melkić and Čavlek 2020).

The other concern of tourists while performing enquires and booking is data ownership, use, and control resulting from the digitization of tourists’ information (Line et al. 2020). Addressing tourists’ privacy concerns, potential hacks, and identity theft are all critical for the sector (Tyan et al. 2020). Blockchain technology can address these challenges since privacy and data protection are built into the blockchain system from the inception of the system’s design (Tyan et al. 2020). Data platforms built on permissioned blockchain give data access only to authorized actors by verifying their identity using a digital signature or certificate (Baralla et al. 2021). Blockchain provides more control and ownership for tourists of the personal data they share with service providers (Kwok and Koh 2019; Line et al. 2020).

The data shared by tourists through blockchain can be used by service providers (with tourist consent) to create mutually beneficial outcomes for both parties, such as personalized products and services based on a complete picture of consumer preferences (Irannezhad and Mahadevan 2021; Line et al. 2020). Blockchain technology could link the complex system of data points that form consumers’ online identities (Tyan et al. 2020; Line et al. 2020). It was evident that blockchain consumers are more comfortable receiving personalized products and services at their discretion and control (Line et al. 2020). Blockchain-powered tourism crowdsourcing platforms accumulate, centralize and process large volumes of tourism data to provide multiple value-added artificial intelligence-based services in real-time, including personalized recommendations and emerging trends for prospective tourists (Leal et al. 2021). Further, knowledge of tourist preferences allows service providers to bundle several types of travel and tourism services such as accommodation, airline, cruise, and railway booking (Irannezhad and Mahadevan 2021).

Also, blockchain enables peer-to-peer (P2P) transactions (e.g., customer to customer) for online bookings and reservations (e.g., hotel reservations; air tickets; tour packages) and permits tourists to use digital (crypto) currency (e.g., Bitcoin, Ripple) without being dependent on a financial institution or relying on third parties or other intermediaries (Valeri and Baggio 2021). The advantage of using crypto payments over conventional payments such as credit cards include non-existent or very low transaction fees and commissions (Melkić and Čavlek 2020), instant transfer without any time lag compared to 3 to 5 business days for credit cards (Willie 2019), no maximum limit on transaction amount (Melkić and Čavlek 2020), and elimination of currency exchange rate concerns for cross-border remittance (Nam et al. 2021; Çapar 2020). Some travel companies have started to list their prices in cryptocurrencies such as Bitcoin, making the payment process easier (Rashideh 2020). Of course, the high volatility of cryptocurrencies is a key barrier to wide adoption across the sector.

Another advantage of blockchain during the pre-travel stage is the execution of smart contracts, which can automate a range of business dealings or transactions between tourists and service providers without human intervention from a legal perspective (Irannezhad and Mahadevan 2021). Smart contracts can be self-executing and self-enforcing by nature as they essentially entail a coded program of an agreement between two or more parties (Tyan et al. 2020). Again, this can create mutually beneficial outcomes for tourists and service providers. It eliminates the need for intermediaries (e.g., notaries, banks, brokers, agents, or other companies), thereby reducing costs and processing times (Ozdemir et al. 2020). Rules, procedures, and penalties are defined and agreed upon by the parties involved in the smart contract (Willie 2019). It enables greater flexibility for tourists, which otherwise was not available with traditional transactions. For instance, with smart contracts, tourists can resell, trade, or exchange bookings such as airline tickets with other travelers if they cannot travel due to any circumstances (Önder and Gunter 2020; Irannezhad and Mahadevan 2021). Some smart contracts allow tourists to rebook if prices decrease after purchase, and the price difference is credited instantly to the tourist’s account (Önder and Gunter 2020). Similarly, smart contracts allow easy cancellations, upgrades, or changes in bookings (Melkić and Čavlek 2020; Kizildag et al., 2019).

Finally, tourist reviews and ratings of service providers (e.g., airlines, cruise, hotels, restaurants) are critical factors in purchasing a tourism product or service (Melkić and Čavlek 2020). Although it has become popular, it has been lambasted for its inability to distinguish between genuine and falsified reporting and trustworthiness (Kizildag et al., 2019; Erceg et al. 2020). Fake reviews, which can become recurring due to ever-increasing business competitiveness, represent a critical issue for tourism (Filimonau and Naumova 2020). The sector needs to ensure that the content of the reviews and ratings published on digital platforms is honest and trustworthy to ensure that consumers can rely on them when researching and planning their travels (Leal et al. 2021). Blockchain can improve the trust and transparency of the online review system (Karode et al. 2020). The immutability of records in blockchain ensures that any review posted on the blockchain platform cannot be deleted, and updates are only possible with a traceable history (Karode et al. 2020). Secure digital identification can ensure authentic customer reviews are distinguishable from inauthentic ones through traceable identities (Filimonau and Naumova 2020). This traceability does not necessarily mean that personal identities have to be revealed; all entries are signed with a unique private key that confirms that a specific transaction comes from a particular user. As a result, users would be unable to create duplicate reviews with the same identity and could impede the manipulation of reviews (Önder and Treiblmaier 2018). Tourists, therefore, can use these trusted rankings and ratings to select service providers.

#### During the trip

Tourists go through numerous identification and registration processes during their journey. For instance, they must carry and present identity documents for airport security, immigration, boarding gate, duty-free shopping, at a hotel, or securing a rental car (Irannezhad and Mahadevan 2021). Digital identification using blockchain technology could improve the way travelers are identified during their journey and avoid unpleasant encounters at various checkpoints. These unique digital IDs can replace passports and all identification-related documents such as birth certificates and driver’s licenses (Rashideh 2020; Önder and Gunter 2020). For instance, blockchain-powered digital identities such as e-passports or digital passports can minimize the waiting time of tourists at border control, especially when combined with smart gates and scanners through automated check-in/out (Nam et al. 2021; Thees et al. 2020). Blockchain-based identity systems (e.g., secure biometric identity systems) could potentially negate this issue of lost passports and reduce identity theft and fraud (Nam et al. 2021; Tyan et al. 2020). Blockchain facilitates quick, automated, and seamless identification of tourists throughout their journey (Melkić and Čavlek 2020; Thees et al. 2020). Also, digital IDs enable archiving medical tourist health records in a secure digital infrastructure that can be accessed from anywhere in the world for those authorized by the patient (Çapar2020). The secure medical archives enable fast access to medical tourists’ history, enabling correct diagnosis and treatment. It can also provide evidence for possible malpractice cases at medical tourist destinations (Çapar 2020).

Blockchain-enabled smart contracts can instantly process travel insurance claims in case of flight delay or cancellation, thereby greatly enhancing the tourism experience (Tyan et al. 2020; Irannezhad and Mahadevan 2021). For instance, AXA, a leading French insurance group, has developed a blockchain platform called Fizzy on the Ethereum platform that offers automatic flight delay insurance for its customers; as soon as a flight delay is detected, compensation is initiated immediately and securely, thus avoiding the need for additional paperwork (Radanović and Likić 2018).

The use of blockchain-enabled digital payments and cryptocurrencies makes cross-border transactions easier and minimizes the risk of foreign currency exchange rates (Treiblmaier et al. 2020). An increasing number of tourism vendors have started accepting cryptocurrencies (Treiblmaier et al. 2020). Importantly, it eliminates credit card thefts (a significant concern for tourists) during travel (Rashideh 2020). During their travels, tourists can use cryptocurrency to pay for admission tickets, souvenirs, public transport fares, ride-sharing, restaurants, and cafes (Treiblmaier et al. 2020). For instance, as early as 2014, Pattaya and Bangkok in Thailand started accepting bitcoin from tourists due to the widespread ATM and credit card fraud (Irannezhad and Mahadevan 2021). Therefore, tourists who visit Thailand are increasingly looking for traders who accept Bitcoin (Rashideh 2020). Similarly, Travelflex coins can be used in the same way as travelers’ checks. It also enables P2P transactions such as customer-to-customer (C2C) transactions as crypto coins can be sent and received directly between users (Nam et al. 2021). Also, cryptocurrencies are convenient to use as they are typically stored in a prepaid digital wallet installed on a user’s mobile phone (Erceg et al. 2020). The increasing acceptance of cryptocurrency has encouraged governments, especially those relying heavily on tourism, to recognize and support the use of cryptocurrencies and even consider creating their own cryptocurrencies (Kwok and Koh 2019; Rashideh 2020; Thees et al. 2020).

Another tourist concern that can be addressed using blockchain is baggage that is delayed, stolen, damaged, or sent to the wrong destination. Such incidents pose a high cost for the aviation sector and cause negative experiences for tourists. The International Airline and Travel Association estimates that the aviation sector could realize an annual savings of over US$500 million by reducing inefficiencies related to baggage mishandling (Irannezhad and Mahadevan 2021). Blockchain technology could significantly improve baggage handling, especially during international travel, as, in many instances, the tourist’s baggage changes hands more than once during their trip (Rashideh 2020). The blockchain shared distributed ledger could allow for luggage and ownership details to be automatically logged on at various points, making it easier to locate luggage in real-time wherever it may be during transit (Willie 2019; Önder and Gunter 2020). Additionally, it is possible to provide tourists with up-to-date information about the current location of their baggage on their smartphones (Willie 2019). Further, baggage tracking can be linked to smart contracts with airlines or travel insurance firms to automatically trigger compensation pay-out when the shared ledger records the lost, damaged, or delayed baggage information (Calvaresi et al. 2019).

The peer-to-peer nature of the blockchain network encourages tourists to deal directly with suppliers, saving time and money (Rashideh 2020). For example, ‘Beenest’ is a blockchain-based firm that matches accommodation providers with seekers (tourists) and is a competitor to Airbnb (Irannezhad and Mahadevan 2021). Similarly, ‘MeetnGreetMe’ is a blockchain-based global C2C platform connecting international travelers with local guides. Tourists can select from approved local guides (Tham and Sigala 2020).

Blockchain-based loyalty programs are another example of a helpful application of blockchain for tourists (Önder and Gunter 2020). It addresses the limitation of most traditional loyalty programs that are neither transferrable nor can be utilized when purchasing from a third party, leading to low redemption rates and high switching costs (Line et al. 2020). In blockchain-based inter-operable loyalty programs, loyalty points (tokens) can be sold or exchanged with others and could earn or redeem loyalty points across multiple vendors throughout the journey (Nam et al. 2021; Önder and Gunter 2020). It enables different firms in the tourism sector (e.g., airlines, hotels, and car rentals) to consolidate and manage their loyalty programs under one blockchain-based loyalty program (Tham and Sigala 2020), thereby addressing the high fragmentation of countless loyalty points, cards, and earning systems (Thees et al. 2020). Blockchain also enables tourists to earn and use their loyalty points in real-time and not wait for the points to be credited much later, as with traditional programs (Irannezhad and Mahadevan 2021). For example, with ‘Trippki,‘ tourists can use their tokens or loyalty points to pay for a hotel stay, restaurant, etc. (Line et al. 2020). Similarly, ‘Loyyal’ is a blockchain-based platform that improves the interoperability of airline loyalty programs by easing the transfer of points to other airlines, partner hotels, and car rentals (Filimonau and Naumova 2020). Blockchain also facilitates innovative rewards programs through the real-time use of tourist locations to generate location-based privileges such as offers and discounts (Nam et al. 2021). Further, blockchain can be used to reward tourists for their sustainable behavior (e.g., using less energy and water at hotels) (Tyan et al. 2020).

Also, blockchain-based product labeling can significantly enhance the truthfulness of specific product labels (Filimonau and Naumova 2020; Rashideh 2020). All information regarding the product (e.g., the ingredients, when and where it is made, stored, and transported) will be available to tourists within seconds by scanning QR codes to determine whether the product is authentic and reliable (Willie 2019). Accurate food labeling is critical due to the rise in health- and allergen-conscious tourists (Filimonau and Naumova 2020). It also alleviates tourists’ concerns with religious considerations such as halal compliance for Muslim tourists (Filimonau and Naumova 2020). Similarly, end-to-end supply chain traceability solutions using blockchain can tackle counterfeit, fake, and contaminated goods. For instance, food-related health and safety is a main concern of tourists. Blockchain solutions can track the food supply at each stage of its journey, from its origin until it reaches the end customer, i.e., from farm to fork, thereby avoiding food contamination and poisoning (Önder and Gunter 2020). If the food supply arrives in bad condition, the offending incident can be tracked precisely, including detailed information on storage temperature, humidity, and GPS data (Baralla et al. 2021; Önder and Gunter 2020). Overall, product labeling and supply chain traceability can address the growing demand of tourists to have food that is organic, local, authentic, sustainably sourced, safe, clean, fresh, and nutritious (Nam et al. 2021; Willie 2019). Similarly, blockchain solutions can tackle drug counterfeiting, a potential problem affecting medical tourists (Önder and Gunter 2020).

#### Post-trip stage

Blockchain technology can encourage tourists to post comments, opinions, pictures, reviews, and feedback about their post-trip experience as they receive crypto coins/tokens as rewards in exchange (Nam et al. 2021). Tourists can use these reward coins/tokens for booking their next trip or convert them into cash or exchange them with other types of cryptocurrencies (Nam et al. 2021). Also, to promote the quality of the review contents, the platform can enforce rules to link rewards to the review acceptance (high rewards for high approval and vice versa) by other users on the platform (Karode et al. 2020). The posted reviews can be marked as “fake” or “genuine,“ or “high quality” or “low-quality” when the majority of users agree. As mentioned earlier, since blockchain uses secure digital identification (unique private key for each identity) with several independent verification processes, the tourist reviews in a tamper-resistance blockchain platform could be considered more reliable and trustworthy and prevent fake and duplicate reviews (Önder and Treiblmaier 2018; Filimonau and Naumova 2020). For example, only reviews posted via verified personal profiles are marked as trusted reviews and monetized for tokens (Karode et al. 2020). Overall, trusted post-trip reviews of tourists will significantly impact the decision-making behavior of potential tourists.

Similarly, blockchain platforms can change the way data are collected in the reflective post-trip phase. Tourists are rewarded for sharing or authorizing the use of their post-trip data with service providers. This data could include transaction history, locations visited, events attended, and activities engaged from previous trips (Line et al. 2020; Nam et al. 2021). Smart contracts could execute the transaction by autonomously collecting or sharing the data with the requesters (tourism service providers). At the same time, the smart contract would transfer the money (or other incentives) to the tourists (Line et al. 2020). Also, based on the shared data, tourists may receive personalized recommendations, bundle offers, and discounts for their next trip (Leal et al. 2021). For example, ‘Travel Chain’ provides a blockchain platform that empowers travelers to share information such as their travel purchases, location, and stays in accommodations in exchange for tokens, which can be used for booking their next flights, hotels, or renting a car (Line et al. 2020). Finally, tourists can receive tokens as rewards for referring other tourists. For example, ‘Travel Block’ provide tourists 5% discount as a referral reward for their next booking (Nam et al. 2021).

## Implications and concluding remarks

The goal of this viewpoint article was to identify and integrate the scattered knowledge on blockchain in tourism into a meaningful and managerially relevant framework. The simplified yet comprehensive conceptualization of blockchain solutions and applications in tourism is critical for the progress of the field, given that the scientific contours of blockchain in tourism are not clearly defined. We expect practitioners and policymakers to find this framework useful for assessing the current and potential blockchain applications in the tourism sector.

Given the proposed framework’s conceptual comprehensiveness and generic nature, we hope that researchers in different countries could adopt, enhance, and use it in their respective contexts. The multitude of uses and applications of blockchain across the different stages of the tourist experience identified in the framework provides a foundation for future studies. Understanding the full potential of blockchain is important now, given its role in supporting the tourism sector to recover from the COVID-19 pandemic. We endeavored to write this viewpoint in an accessible way with the hope that it is useful for practitioners and policymakers to familiarize themselves with blockchain applications and the benefits afforded by the technology.

The use of blockchain technologies in tourism can potentially bring down the overall cost structure and benefit tourists and various service providers in the sector. The decrease in costs will boost the sector, enabling price-sensitive people to undertake travel, especially in these economically challenging times brought about by the COVID-19 pandemic. Further, a reduction in manual processing and paper transactions will also prove helpful during the pandemic when people feel uncomfortable about face-to-face contact and handling paper. Blockchain can enhance the overall user experience in the tourism sector by increasing transparency and access to up-to-date information, reducing costs, and minimizing the number of transactions and the need for intermediaries. Blockchain can increase opportunities for entrepreneurs in the tourism sector, whereby small tour operators and accommodation providers can set up shop and establish credible identities through blockchain platforms. The trust accorded by blockchain will enhance user (tourist) trust in using these entrepreneurial ventures’ services and help expand the tourism market. Another benefit from the user perspective is that the use of blockchain shifts the ownership of data to users, thereby reducing the unilateral usage of customer data by service providers for marketing purposes or other activities that may result in financial gain to the provider without customer knowledge (Line et al. 2020). Moreover, blockchain can improve the coordination between stakeholders. For example, if a tourist does not check in for their flight, this can trigger an update of the car rental company’s inventory and the hotel’s availability (Treiblmaier 2020). Overall, the insights and the framework are helpful for practitioners and policymakers looking to take advantage of the full opportunities of blockchain in tourism.

Given the uncertainty over how long this COVID-19 pandemic will last, with several countries experiencing or likely to experience the new wave of infections due to evolving COVID-19 variants, we anticipate the uptake of blockchain technology in the tourism sector to continue to accelerate. However, several factors limit the adoption of blockchain technology in the tourism sector. For instance, many countries have not developed a regulatory framework that legitimizes the use of cryptocurrency, which affects users’ trust (Treiblmaier et al. 2020). Further, this legal vacuum could also lead to inappropriate use of cryptocurrencies, such as money laundering and funding terrorists or other illegal activities (Treiblmaier et al. 2020). Many blockchain applications rely on technical know-how, attitude towards trying out new technology, and access to mobile devices. There is still a digital divide between developed and underdeveloped nations (Sigala 2020). A significant proportion of the world population does not have the devices or the infrastructure to access the required technologies. This divide may prevent some countries and small businesses from reaping the benefits of blockchain-enabled technologies. The other concern of blockchain is the lack of system scalability, such as adapting to the increased data processing requirements (Melkić and Čavlek 2020). In addition, the low transaction speed and high energy consumption of blockchain could hinder the widespread implementation of blockchain solutions (Ozdemir et al. 2020).

Although we have presented a comprehensive blockchain framework for tourism, it still may not have covered every blockchain solution and application in tourism. Still, we are confident that the insights offered, including the framework, contribute towards advancing both theory and practice and offer pragmatic and managerially relevant blockchain-enabled solutions that could facilitate the widespread implementation of blockchain in the tourism sector. We are optimistic that our framework will encourage and help guide future research on this important domain.
